# Notch1-ADAM8 positive feed-back loop regulates the degradation of chondrogenic extracellular matrix and osteoarthritis progression

**DOI:** 10.1186/s12964-019-0443-2

**Published:** 2019-10-22

**Authors:** Biao Duan, Yan Liu, He Hu, Fu-Guo Shi, Ya-Long Liu, Hao Xue, Xin-Yu Yun, Ming-Yu Yan, Xi-Rui Han, An-Fu Chen, Yong Wang, Zhe-Hai Li

**Affiliations:** 1 Reproductive Center, Ganzhou People’s Hospital, No.17 Hongqi Avenue, Zhanggong District Ganzhou, 314000 People’s Republic of China; 2 Inner Mongolia Medical University, Jinshan District, Hohhot, 010110 People’s Republic of China; 3 Department of Orthopedics, Inner Mongolia Medical University Third Affiliated Hospital, No.20 Shaoxian Road, Kundulun District, Baotou, 014000 People’s Republic of China; 4 Department of Orthopedics, Inner Mongolia People’s Hospital, No.20 Zhaowuda Road, Saihan District, Hohhot, 010017 People’s Republic of China; 5 Department of Chinese medicine, Preventive health center of Baotou steel group, Aerding Street, Kundulun District, Baotou, 014000 People’s Republic of China; 6 Department of Orthopedics, Yangling Demonstration District Hospital, No.8 Houji Road, Yangling District, Xianyan 712100 People’s Republic of China; 7 Department of Pediatric Orthopedics, Fourth Hospital of Baotou, Aogen Road, Qingshan District, Baotou, 014010 People’s Republic of China; 8 Department of Orthopedics, Peking University Third Hospital, No.10 Courtyard, Chedaogou, Haidian District, Beijing, 100083 People’s Republic of China

**Keywords:** Notch1, ADAM8, ECM, MMP-9, Osteoarthritis

## Abstract

**Background:**

Osteoarthritis (OA) is one of the most prevalent joint disease, and there are still no effective therapeutic agents or clinical methods for the cure of this disease to date. The degradation of cartilage extracellular matrix (ECM) is a major cause of OA.

**Method:**

IL-1β was used to induce chondrogenic degradation. Q-PCR and Western blotting were used to detect mRNA and protein level, respectively. ELISA was used to detect the secreted TNF-α and IL-6 level. Immunofluorescence was used to detect the protein level of Aggrecan, Collagen II and ki67. TUNEL and flow cytometry were used to examine cell apoptosis of chondrocytes. ChIP and luciferase assay were used to study molecular gene regulation. Osteoarthritic animal model and Safranin-O staining were used to determine the in vivo OA phenotype.

**Results:**

The expression of ADAM8 was up-regulated in osteoarthritic chondrocytes. Knockdown of ADAM8 suppressed the OA phenotype in the in vitro OA cell model. ADAM8 regulated OA progression through the activation of EGFR/ERK/NF-κB signaling pathway. Inhibition of Notch signaling suppressed OA phenotype in the in vitro OA cell model. Notch signaling regulated the gene expression of ADAM8 directly via Hes1. Notch1-ADAM8 positive feedback loop promoted the progression of OA in vivo.

**Conclusion:**

Notch1-ADAM8 feed-back loop regulates the degradation of chondrogenic extracellular matrix and osteoarthritis progression.

## Background

Osteoarthritis (OA) is one of the most prevalent joint disease, affecting 25% of the adult population in the whole world [1]. Typically, progressive loss and destruction of articular cartilage, formation of osteophytes, inflammation of the synovium were found in the OA joints [2]. Due to the limited understanding of the pathophysiology of OA, there are still no effective therapeutic agents or clinical methods for the cure of this disease to date [3]. Hence, there is an urgent necessity for the investigations on the molecular and cellular mechanism of OA.

The degeneration of cartilage tissues is a main pathological change in OA joints [4]. The degeneration of cartilage are originated from the degradation of cartilage extracellular matrix (ECM), which composed of majorly type II collagen and aggrecan [5] . The degradation of chondrogenic ECM is conducted by proteases, including matrix metalloproteinases (MMPs) for degrading collagens [6], and Adamalysin with Thrombospondin Motifs (ADAMTS) for degrading aggrecans [7]. The collagenase, including MMP-1, − 9, − 13 and − 14 are found to play crucial role for the degradation of collagens [8]. MMP-13 is thought to be the primary and an important collagenase in OA, with its expression increased in OA cartilage [9]. MMP-9 is found to be a crucial ECM degradation enzyme, since the expression of MMP-9 was up-regulated in OA [10], and the knockout of *MMP-9* gene in mice resulted in a significant reduction of OA phenotype in osteoarthritic animal model [11]. ADAMTS4 and ADAMTS5 are considered as two of the most critical matrix degradation enzymes. Evidence showed that the knockout of *ADAMTS5* resulted in less severe cartilage damage in a murine surgical model of OA and in an antigen-induced arthritis model [12, 13]. More studies indicated that ADAMTS4 plays a crucial role in human OA [14, 15]. Furthermore, the inhibition of ADAMTS4 or ADAMTS5 by specific inhibitors resulted in attenuated severity of OA symptoms [16–18].

In addition to MMPs and ADAMTS, A Disintegrin and Metalloproteinase (ADAM) family is considered to be involved in mediating ECM degradation in OA [19]. Several of the ADAM proteins are found to be up-regulated in OA. For example, ADAM10 was found to be notably up-regulated during the progression of OA, and was confirmed to be stimulated by inflammatory factor IL-1, and was considered to be involved in cartilage degeneration [20, 21]. ADAM9, ADAM12, ADAM15, ADAM19 and ADAM23 were also found to be up-regulated in human OA cartilage [22–24]. Despite that the investigations of ADAM8 are majorly from the research area of tumorigenesis [25, 26], the role of ADAM8 in OA has been included in several studies. As identified by several studies, the expression of ADAM8 was found to be elevated in OA cartilage [24, 27, 28]. Importantly, Zack. MD *et.al* indicated that ADAM8 is a fibronectinase in human OA chondrocytes, it cleaves fibronectin at the amino acid Ala (271) [28]. This ADAM8 mediated degradation of fibronectin in cartilage may contribute to OA progress directly. Besides, ADAM8 was identified to be a regulator in rheumatoid arthritis associated osteoclastogenesis and bone erosion [29–31]. However, the exact role of ADAM8 in regulating the degradation of ECM, including collagens and aggrecans, and in modulating OA progression is still to be elucidated.

Notch signaling has been found to contribute to OA development [32, 33]. Notch receptors Notch1 and Notch2 are up-regulated in osteoathritic chondrocytes, and these Notch receptors are cleaved to form Notch-intracellular domain (ICD) and translocated to nucleus, thus the Notch signaling is activated, by binding to Rbpj protein to form a transcriptional activator, and inducing Hes/Hey family protein to exert regulatory function [32]. The knockout of Rbpj gene in mice resulted in a suppression of OA development. Furthermore, the inhibition of Notch signaling by specific inhibitor led to a protection of the mouse joint from developing OA phenotype [32]. More studies indicated that, Hes1, the downstream effector of Notch signaling was highly expressed in OA chondrocyte [33]. Hes1 was induced by the activated Notch receptor ICD, and induced strong expression of MMP13, which catalyzed the degradation of chondrogenic ECM, and resulted in a degeneration of cartilage [32]. Therefore, the Notch-Hes1 axis is considered as an important mechanism for OA development.

In the present study, we investigated the role of ADAM8 in the regulation of OA development. Our results indicated that in OA, the expression of ADAM8 was stimulated by Notch1-Hes1 cell signaling pathway. The promotion of ADAM8 facilitated the development of osteoarthritic phenotype of chondrocytes, and resulted in the promotion of OA progression. Furthermore, ADAM8 was found to facilitate the activation of Notch1 protein, in turn to stimulate ADAM8 gene expression, thus to form a positive-feedback mechanism.

## Methods

### Cell isolation and culturing

Rat chondrocytes were isolated from the cartilage of knee articular joints. Cartilage was cut and sectioned to small pieces, and then was treated with 0.25% Tripsin (Invitrogen) for 1 h and was treated with type-II collagenase for 5 h. The isolated chondrocytes were maintained with DMEM medium (Hyclone), supplemented with 10% fetal bovine serum (FBS) (Hyclone) and antibiotics (1% penicillin and streptomycin) (Invitrogen). Chondrocytes were passed to next generation when cells reached each other. Generation passage 1 to 4 was used for tests in experiments.

### IL-1β induced inflammation of chondrocytes

Chondrocytes were pre-seeded in 6 well plates and maintained with growth medium for overnight culture. IL-1β was added to the cell to induced OA-like inflammation of chondrocytes. For the inhibition of Notch signaling, MW167 (Sigma-Aldrich) was used to treat chondrocytes. For the inhibition of ERK signaling, U0126 (Sigma-Aldrich) was used to treat chondrocytes.

### RNA extraction and real-time PCR

Total RNA from cultivated chondrocytes or animal tissues was isolated by TriZol reagent (Thermo Fisher Scientific), according to the manufacturers’ instructions. cDNA was synthesized from total RNA with a High Capacity cDNA Reverse Transcription kit (Thermo Fisher Scientific). Real-time PCR was performed with a SYBR green Premix Ex Taq reagent kit (Kakara) on a thermocycler (7500, Applied Biosystems, USA). The relative expression levels were calculated using the 2^-∆∆Ct^ method and normalized with GAPDH as the internal reference.

### Western blotting

The reagent RIPA Lysis Buffer (Thermo Fisher Scientific) was used to extract the total protein of the cultivated chondrocyte or isolated cartilage tissue. RIPA was supplemented with protease inhibitor PMSF. Protein concentration was determined by a BCA kit (Thermo Fisher Scientific). Protein was mixed with protein loading buffer (Thermo Fisher Scientific) and loaded to a 12% SDS-PAGE gel for electrophoresis. Afterward, the gel was transferred to PVDF membrane (Thermo Fisher Scientific). After a blocking step with a blocking buffer, the membrane was incubated with primary monoclonal antibodies at 4 °C overnight. After washing, the membrane was incubated with secondary antibody. Finally, signal was detected by an ECL method. The primary antibodies used in this study includes anti-Aggrecan (1:1000, Abcam), anti-Collagen II (1:1000, Abcam), anti-β-actin (1:3000, Abcam), anti-ADAM8 (1:1000, Abcam), anti-p-ERK (1:1000, Abcam), anti-ERK (1:2000, Abcam), anti-p- NF-κB p65 (1:1000, Abcam), anti-NF-κB p65 (1:2000, Abcam), anti-MMP9 (1:1000, Abcam), anti-Notch1 (1:1000, Abcam), anti-Hes1 (1:2000, Abcam). All the primary antibodies were incubated for 4 h at 37 °C. The HRP-conjugated secondary antibody (1:5000, Sigma-Aldrich) was incubated for 2 h at RT.

### Gene knockdown and overexpression

Gene knockdown or overexpression were performed by infection of Adeno-viruses which carry overexpression plasmids or shRNA plasmids. The adeno-viruses Ad-ADAM8, Ad-sh-ADAM8 and Ad-sh-EGFR were customized ordered from Shanghai Hanheng Biotech. Ltd. The infection of viruses to chondrocytes was mediated by LipofectamineTM ^2000^ (ThermoFisher, USA) according to the manufacturer’s instructions.

### Elisa

The amount of chondrocyte secreted TNF-α and IL-6 were determined by an ELISA kit (Biocompare, USA), according to the manufacturer’s instruction.

### Immunofluorescence staining

The protein level of Aggrecan, Collagen II and ki67 in chondrocyte were examined by immunofluorescence staining. Chondrocytes were seed on the glass plates in 6 well plates. At the indicated time point, chondrocyte was fixed with 4% PFA for 1 h, then treated with 0.5% Triton X-100 for 10 min. Next step, the cells were treated with 1% BSA to reduce background. Afterward, primary and secondary antibodies were used, and nucleus was stained with DAPI. Samples were observed by Leica microscopy.

### ChIP assay

Chromatin immunoprecipitation (ChIP) assays were performed via a chromatin immunoprecipitation kit (Millipore, Temecula, CA). The antibody anti-Hes1 (Abcam) was used. Chondrocytes were first cross-linked by formaldehyde at a final concentration of 1% for 10 min. Then chondrocytes were washed twice with cold PBS (with protease inhibitors), collected and resuspended in 200 μl SDS lysis buffer (1% SDS, 10 mM EDTA, 50 mM Tris-HCl, pH 8.0), then was incubated for 10 min on ice. The lysates were then sonicated for 5 times. Next, the samples were centrifuged, and the supernatants were collected and diluted 10-fold in ChIP dilution buffer. Cross-linked chromatin was incubated overnight with 15 μg anti-Hes1 antibody or control IgG in a total volume of 1 ml at 4 °C. Antibody-protein-DNA complexes were immunoprecipitated by 60 μl salmon sperm DNA/protein A. Pellets were washed and eluted by elution buffer (1% SDS, 0.1 M NaHCO3). 5 M NaCl was used for the reverse of cross-linking. Samples were purified through QIAquick PCR purification kit (Qiagen, Chatsworth, CA). Q-PCR amplifications were performed using the purified DNA and ChIP primers of ADAM8 promoter.

### Luciferase assay

Dual luciferase reporter system (Promega) was used for luciferase assay. ADAM8 promoter WT or mutant sequences were inserted to the plasmid pGL3 vector. HEK293T cells were seeded at 4 × 10^4^ cells/well, and each well was transfected with a total of 400 ng of pGL3 vector or pGL3-ADAM8 WT, or pGL3-ADAM8 Mutant, was transfected by lipofectamineTM ^2000^ (ThermoFisher, USA) 24 h after seeding. Cells were co-transfected with pcDNA3.1-Hes1 or shRNA-Hes1, both of which were customized ordered from Shanghai Hanheng Biotech. Ltd. Cells were lysed 24 h after transfection and luciferase activity was measured for each well by Dual-luciferase reporter assay system (Promega).

### TUNEL staining and flow cytometry

All steps were according to the instructions of TUNEL kit (Keygen Biotech, Nan jing, jiangsu, China). Briefly, chondrocytes were seeded on the 24 well plates with the cell density 2 × 10^5^ cells per well, incubated with complete growth medium (DMEM, 10% FBS and 1% Penicillin-Streptomycin). After the indicated duration of incubation and treatments, medium was removed, and cells were fixed with formaldehyde. Next, cells were incubated with Triton X-100 for 5 min at room temperature. After wash with PBS, cells were incubated in TUNEL staining solution and incubate away from light for 60 min at 37 °C. Then, after wash again by rinse buffer, cells were developed and evaluated with a fluorescence microscope. The apoptotic rate was quantified with the TUNEL positive rate.

Cell apoptosis was analyzed by flow cytometry using an Annexin V-fluorescein isothiocyanate (FITC)/propidium iodide (PI) Apoptosis Detection kit (Sigma-Aldrich) according to the manufacturer’s protocol. After cell samples fixed, FITC-Annexin V and PI were added for 20 min in darkness at room temperature. Subsequently, Annexin V binding buffer was added to the mixture prior to quantifying the fluorescence using a FACSort flow cytometer. Cell apoptosis was analyzed using CytExpert version 2.3.

### Animal experiments

In total, 36 male SD rats, weight ranging from 150 to 200 g, were purchased from Hunan Slake Jingda Experimental Animal Co. Ltd., China. Rats were randomly assigned to six groups: control (*n* = 6), OA operation (n = 6), OA operation+MW167 (n = 6), OA operation+Ad-ADAM8 (n = 6), OA operation+Ad-sh-ADAM8 (n = 6). The rat OA model was established by partial removal of the medial meniscus of the knee as previously described [34] . Briefly, after 1% sodium pentobarbital anesthesia and iodine sterilization, the surgery was performed. A lateral parapatellar skin incision was made longitudinally in the knee joint at a level of 1 cm proximal to the patella. The medial meniscus and tibial ligament were disconnected, then partial medial meniscus was removed. The patella was then placed in the original position and sealed with a scalpel. For the control, sham operations were performed with only lateral parapatellar skin incision was made and sealed with a scalpel. After the suture, the limb was not fixed for free movement, and penicillin was sometimes injected appropriately to prevent infection. At 12 weeks after surgery, cartilage tissues were isolated and sectioned, then immunohistochemistry, Safranin-O staining and toluidine blue staining were performed. Sections were fixed with 4% paraformaldehyde for 15 min at 25 °C, 2 times of washes with PBS, and followed by incubation of primary and secondary antibodies for IHC, or stained with Safranin-O solution, or 0.5% (W/W) toluidine blue for 30 min at 25 °C.

### Statistical analysis

Data are expressed as mean ± standard deviation (SD) values based on at least 3 replicates independently. Comparisons between more than two groups were analyzed by one-way ANOVA followed by Tukey post hoc tests. Comparisons between two groups were made with two-tailed t-tests. Statistical analysis was performed using SPSS 10.0 for Windows. *P* < 0.05 was considered statistically significant.

## Results

### The expression of ADAM8 was up-regulated in osteoarthritic chondrocytes

**Fig. 1:**
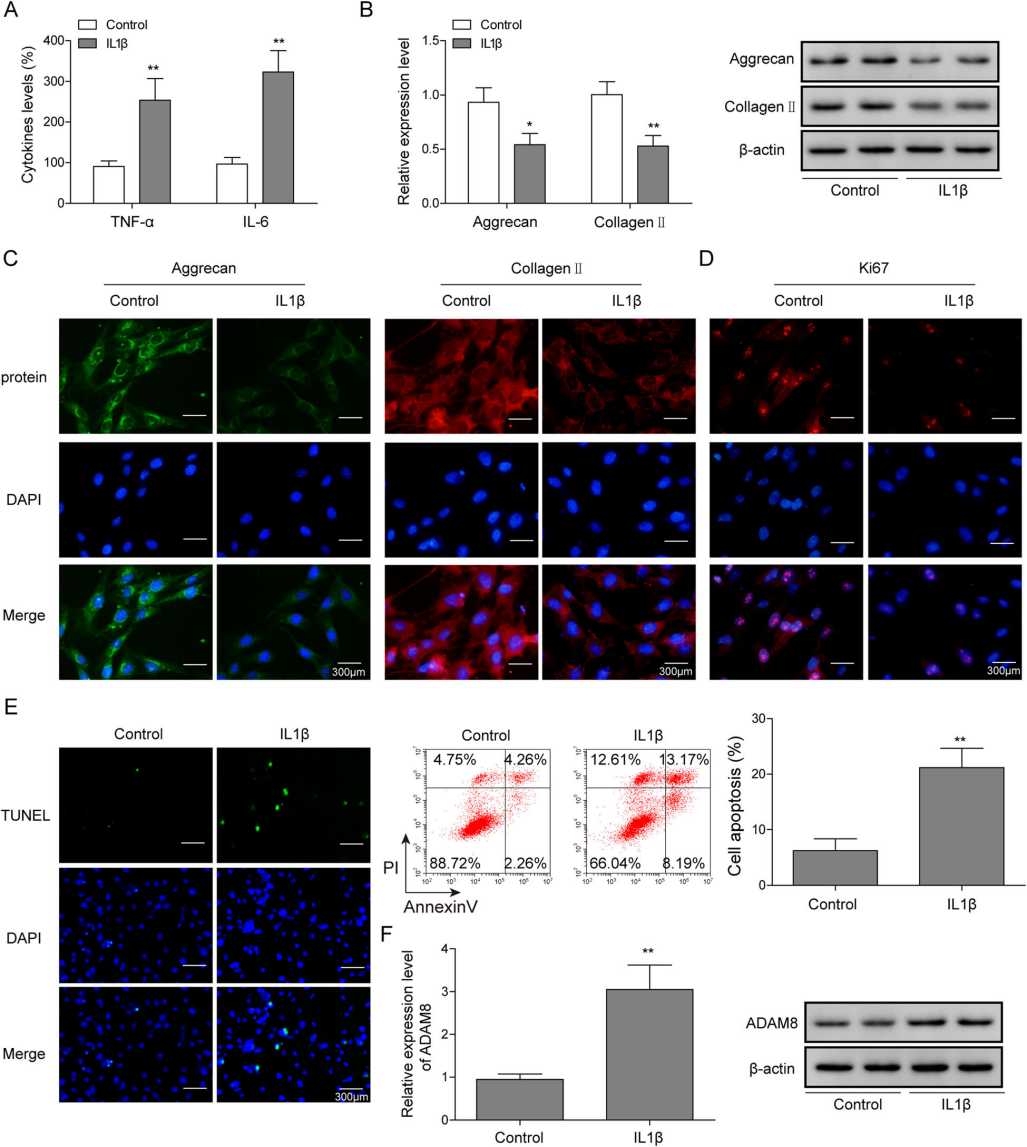
The expression of ADAM8 was up-regulated in osteoarthritic chondrocytes. **a**: The protein level of TNF-α and IL-6 were measured by ELISA. Results indicated that the secreted TNF-α and IL-6 were promoted in OA chondrocytes. **b**: the protein level of Aggrecan and Collagen II were measured by Western blotting. Results indicated that the expression of Aggrecan and Collagen II were suppressed in OA chondrocytes. **c**: the suppressed expression of Aggrecan and Collagen II was confirmed by immunofluorescence staining method. **d**: immunofluorescence results indicated that the expression of ki67 was suppressed in OA chondrocytes, suggesting that cell proliferation was suppressed. **e**: flow cytometry results indicated that cell apoptosis of OA chondrocytes was promoted. **f**: mRNA and protein level of ADAM8 were up-regulated in OA chondrocytes, as indicated by Q-PCR and western blotting

To explore the gene expression of ADAM8 in osteoarthritic chondrocytes, an in vitro OA cell model was established. In this model, the primary rat chondrocytes were treated with IL-1β. In this cell model, the OA-like phenotype was detected after IL-1β treatment. As shown in Fig. 1a, ELISA results indicated that the production of TNF-α and IL-6 was found to stimulated in OA chondrocytes, compared to the negative control. Both of Q-PCR and western blotting results revealed that the expression of chondrogenic markers, including the Collagen II and Aggrecan, were found to be suppressed by IL-1β, suggesting that the expression of chondrogenic ECM was inhibited (Fig. 1b). As indicated by Fig. 1c, the protein level of these two protein markers were confirmed to be down-regulated via an immunofluorescence assay, suggesting that there was a degeneration of chondrogenic ECM in this cell model. As shown in Fig. 1d, there was a decline of Ki-67 protein level found in the OA chondrocytes, suggesting that the proliferation of IL-1β treated chondrocytes was suppressed. Moreover, a stimulated apoptosis was found in IL-1β treated chondrocytes, as shown in Fig. 1e. These evidences shown in the above data suggested that an in vitro OA cell model was well established. Next, the gene expression of ADAM8 was evaluated. As shown in Fig. 1f, compared to the control, the expression of ADAM8 was significantly promoted in OA chondrocytes, as indicated by both Q-PCR and western blotting results. This result demonstrated an up-regulation of ADAM8 in OA chondrocytes.

### Knockdown of ADAM8 suppressed the OA phenotype in vitro OA cell model

**Fig. 2:**
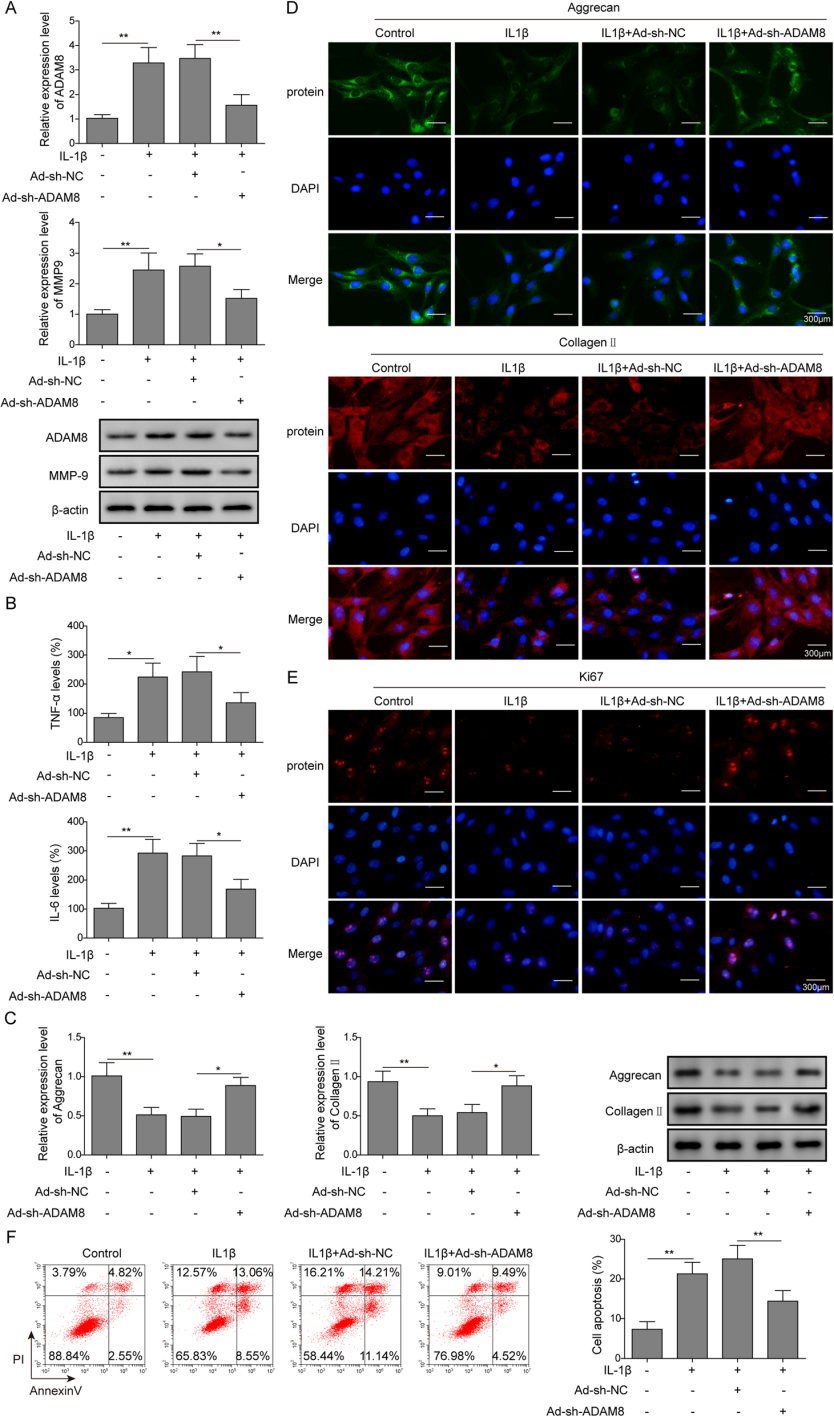
Knockdown of ADAM8 suppressed the OA phenotype in vitro OA cell model. **a**: mRNA and protein level of ADAM8 were down-regulated by the transfection of ADAM8 shRNA; Besides, mRNA and protein level of MMP9 were down-regulated by the treatment of ADAM8 knockdown. **b**: the level of secreted TNF-α and IL-6 were down-regulated by the treatment of ADAM8 knockdown. **c**: the mRNA and protein level of Aggrecan and Collagen II were promoted by the treatment of ADAM8 knockdown. **d**: the up-regulation of Aggrecan and Collagen II by ADAM8 knockdown was confirmed by the immunofluorescence results. **e**: the knockdown of ADAM8 promoted the expression of ki67 in chondrocytes, suggesting that the knockdown of ADAM8 stimulated cell proliferation. **f**: cell apoptosis of chondrocytes was suppressed by the treatment of ADAM8 knockdown, as indicated by flow cytometry assay

To explore the regulatory role of ADAM8 in OA, an artificially knockdown of ADAM8 expression was performed in the in vitro OA cell model, through the transfection of Adv-shRNA-ADAM8. As shown in Fig. 2a, a stimulation of ADAM8 expression by IL-1β was detected by both Q-PCR and Western blotting, however, the expression of ADAM8 was notably attenuated by Adv-shRNA-ADAM8, suggesting that the knockdown of ADAM8 was successful, and the promotion of ADAM8 in OA chondrocytes can be reversed by a shRNA mediated gene silence. Meanwhile, we found that the expression of MMP-9 was stimulated by IL-1β, surprisingly, this promotion of MMP-9 can be reversed by Adv-shRNA-ADAM8 as well. This result suggested that knockdown of ADAM8 resulted in a down-regulation of MMP-9. As shown in Fig. 2b, ELISA experiments revealed that the IL-1β mediated stimulation of TNF-α and IL-6 was found to be blocked by the gene silence of ADAM8, suggesting that the knockdown of ADAM8 resulted in a reduction of TNF-α and IL-6 expression. As shown in Fig. 2c, Q-PCR and Western blotting results indicated that the expression of chondrogenic ECM genes, including *Collagen II* and *Aggrecan*, was suppressed by IL-1β, however, this suppression was reversed by Adv-shRNA-ADAM8. This result was confirmed by immunofluorescence assays, as shown in Fig. 2d. Furthermore, Adv-shRNA-ADAM8 was found to promote the expression of the cell proliferation marker Ki-67 in OA chondrocytes, suggesting that knockdown of ADAM8 reversed IL-1β induced inhibition of cell proliferation of chondrocytes (Fig. 2e). As revealed by Fig. 2f, Adv-shRNA-ADAM8 was found to attenuate cell apoptosis of OA chondrocytes, suggesting IL-1β induced chondrogenic apoptosis was reversed by the knockdown of ADAM8. Collectively, these results demonstrated that the gene knockdown of ADAM8 attenuated OA phenotypes in the in vitro OA cell model.

### ADAM8 regulated OA progression through the activation of EGFR/ERK/NF-κB signaling pathway

**Fig. 3:**
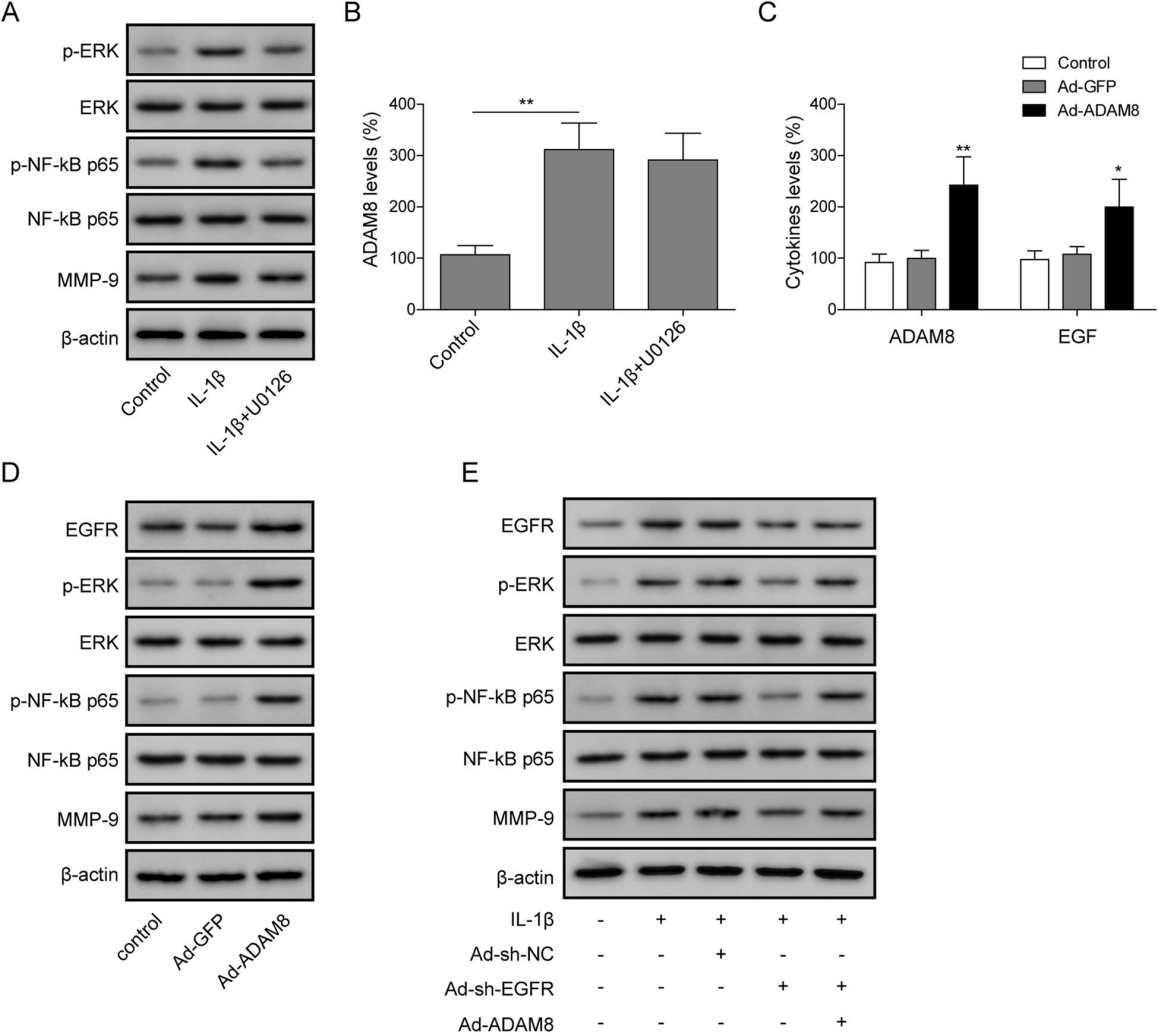
ADAM8 regulated OA progression through the activation of EGFR/ERK/NF-κB signaling pathway. **a**: protein level of ERK, p-ERK, NF-kB p65, p-NF-kB p65 and MMP-9 were measured by Western blotting. Protein level of p-ERK, p-NF-kB p65 and MMP-9 were stimulated by IL-1β, however, were blocked by ERK inhibitor U0126. **b**: the secreted form of ADAM8 was increased by IL-1β, and was not affected by the treatment of U0126. **c**: overexpression of ADAM8 increased the secreted form of ADAM8 and EGF. **d**: Western blotting results indicated that overexpression of ADAM8 stimulated the protein level of EGF, p-ERK, p-NF-kB p65 and MMP-9. **e**: the protein level of EGFR, p-ERK, p-NF-kB p65 and MMP-9 were stimulated by IL-1b, however, were suppressed by the treatment of Adv-sh-EGFR. Additionally, the treatment of Adv-ADAM8 diminished the suppressive effects of Adv-sh-EGFR

In this section of study, we sought to investigate the mechanism for ADAM8 mediated promotion of MMP-9 expression and ECM degradation. According to previous report, Scholmann. U *et. al* pointed out that ADAM8 regulate the pathway ERK1/2 (extracellular signal–regulated kinases 1/2) signaling to affect the expression of MMPs in pancreatic ductal adenocarcinoma cells. Additionally, NF-κB (nuclear factor kappa-light-chain-enhancer of activated B cells) signaling was found to be stimulated in the ADAM8 overexpression osteoclast precursors [35]. Therefore, we tried to confirm the relationship between ADAM8 and ERK/ NF-κB signaling in OA chondrocytes. As shown in Fig. 3a, the protein level of phosphorylated-ERK1/2 (p-ERK1/2) and phosphorylated-p65 (p-p65), as well as MMP-9 were found to be stimulated by IL-1β, suggesting that ERK1/2 and NF-κB signaling were activated by IL-1β. To confirm whether ERK1/2 and NF-κB signaling affects ADAM8 expression, U0126, the ERK1/2 specific inhibitor was used. As shown in Fig. 3a, U0126 blocked the IL-1β mediated activation of p-ERK1/2, p-p65 and MMP-9 protein level, suggesting that U0126 inhibited both ERK1/2 and NF-κB in OA chondrocytes. As shown in Fig. 3b, Q-PCR results indicated that U0126 exerted no significant change to the mRNA level of ADAM8, suggesting that the up-regulation of ADAM8 in OA was not a result of the activation of ERK1/2 and NF-κB signaling. Oppositely, as indicated by Fig. 3d, our results showed that, the overexpression of ADAM8 in chondrocytes led to a strong up-regulation of p-ERK1/2, p-p65 and MMP-9 protein level, suggesting that ADAM8 stimulated ERK1/2 and NF-κB signaling. Previous reports indicated that, as a disintegrin metalloprotease, ADAM8 cleaved membrane anchored CD23 protein [36]. The cleaved CD23 protein was soluble and activated, in turn to act as a regulator to cause up-regulation of IgE production and the induction of inflammatory cytokines [37]. Therefore, we proposed that the secreted form of ADAM8 may act as a proteolytic enzyme to activate putative protein factors to mediate a stimulation of ERK1/2 and NF-κB cell signaling. Due to the fact that EGF-EGFR activates the downstream cell signaling ERK1/2 and NF-κB in various cell types (35–38) [38–41], we speculated that ADAM8 may mediate a promotion of the active growth factor EGF. As shown in Fig. 3c, the overexpression of ADAM8 in chondrocytes resulted in a promotion of secreted form of EGF, as revealed by the ELISA experiment. As shown in Fig. 3d, Western blotting results indicated that, the protein level of EGFR was also stimulated by ADAM8 overexpression. These results suggested that ADAM8 promoted EGF-EGFR cell signaling. As shown in Fig. 3e, IL-1β stimulated the protein level of EGFR、p-ERK、p-p65、MMP-9. However, this IL-1β mediated promotion of protein expression was attenuated by the knockdown of EGFR expression, and this promotion of protein expression was again rescued by ADAM8 overexpression. Collectively, these results suggested that the stimulation of EGF and EGFR, the activation of the downstream ERK1/2 and NF-κB signaling pathway, as well as MMP-9 expression were ADAM8 dependent.

### Inhibition of Notch signaling suppressed OA phenotype in vitro OA cell model

**Fig. 4:**
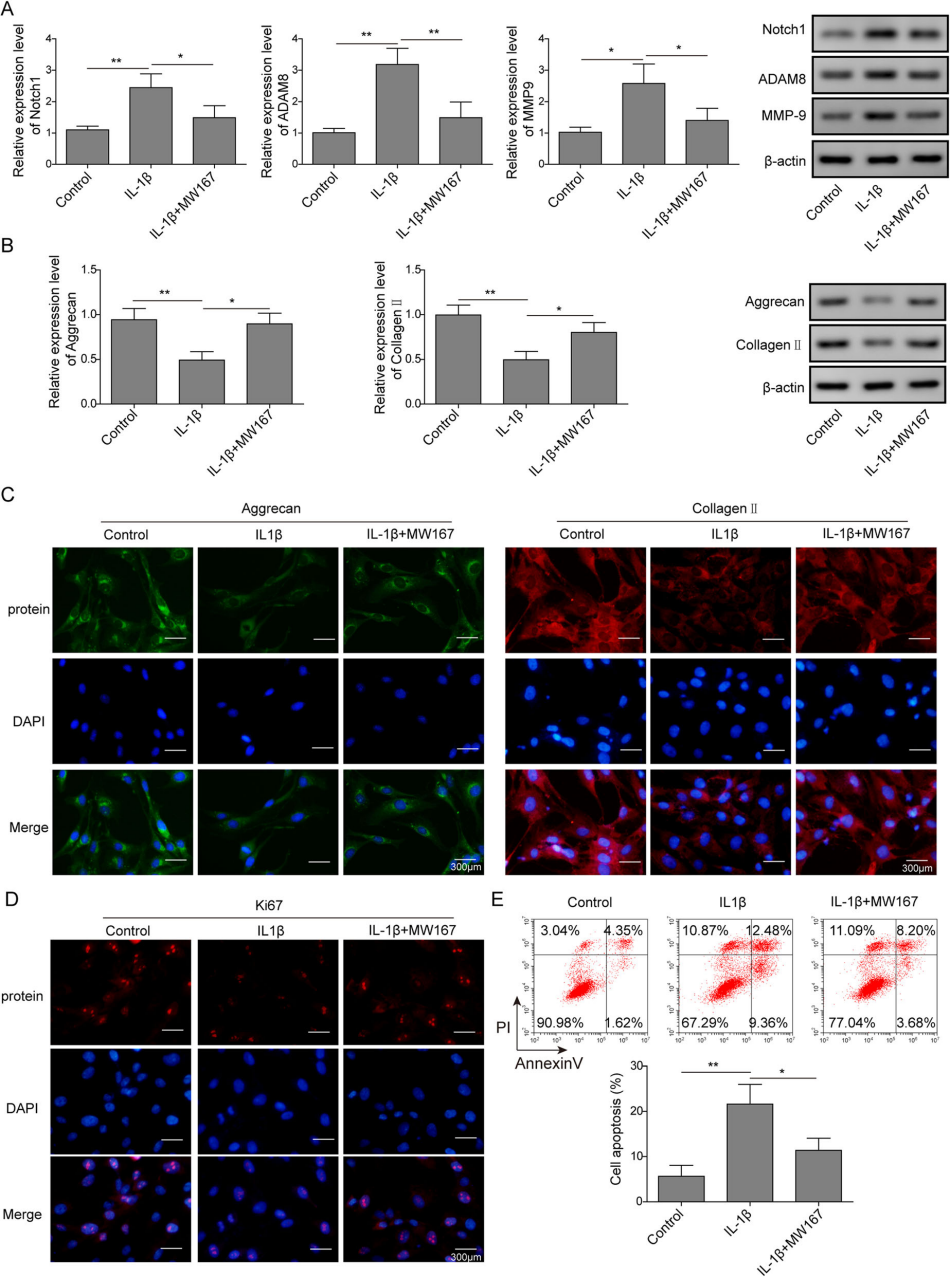
Inhibition of Notch signaling suppressed OA phenotype in vitro OA cell model. **a**: gene expression of Notch1, ADAM8 and MMP-9 were stimulated by IL-1β, however, were blocked by Notch inhibitor MW167. **b**: Western blotting results indicated that the protein level of Aggrecan, Collagen II were suppressed by IL-1β, however, were rescued by the treatment of Notch inhibitor MW167. **c**: immunofluorescence results indicated that the protein level of Aggrecan, Collagen II were suppressed by IL-1β, however, were rescued by the treatment of Notch inhibitor MW167. **d**: immunofluorescence results indicated that the protein level of ki67 was suppressed by IL-1β, however, was rescued by Notch inhibitor MW167. **e**; cell apoptosis of chondrocytes was induced by IL-1β, however, was blocked by MW167

According to previous studies, Notch-Hes1 pathway exerts positive effect for the development of OA [33] . We proposed that Notch-Hes1 pathway may regulate OA development by affecting ADAM8 expression. As shown in Fig. 4a, the Notch inhibitor MW167 was used for the treatment of OA chondrocytes. Q-PCR and western blotting results revealed that the expression of Notch1, ADAM8 and MMP-9 were stimulated by IL-1β, however, the expression of these genes was all attenuated significantly by MW167. As shown in Fig. 4b, Q-PCR and Western blotting results revealed that the expression of Collagen II and Aggrecan were suppressed by IL-1β, however, the expression of these genes was all rescued significantly by MW167. This result was confirmed by immunofluorescence assays (Fig. 4c). As shown in Fig. 4d, immunofluorescence assays results indicated that the expression of Ki-67 was suppressed by IL-1β, however, was rescued significantly by MW167. As shown in Fig. 4e, the flow cytometry results revealed that cell apoptosis of chondrocytes was induced by IL-1β, however, was blocked by MW167 significantly. Collectively, these results demonstrated that Notch1 signaling stimulated the expression of ADAM8 and MMP-9.

### Notch signaling regulated the gene expression of ADAM8 directly via Hes1

**Fig. 5:**
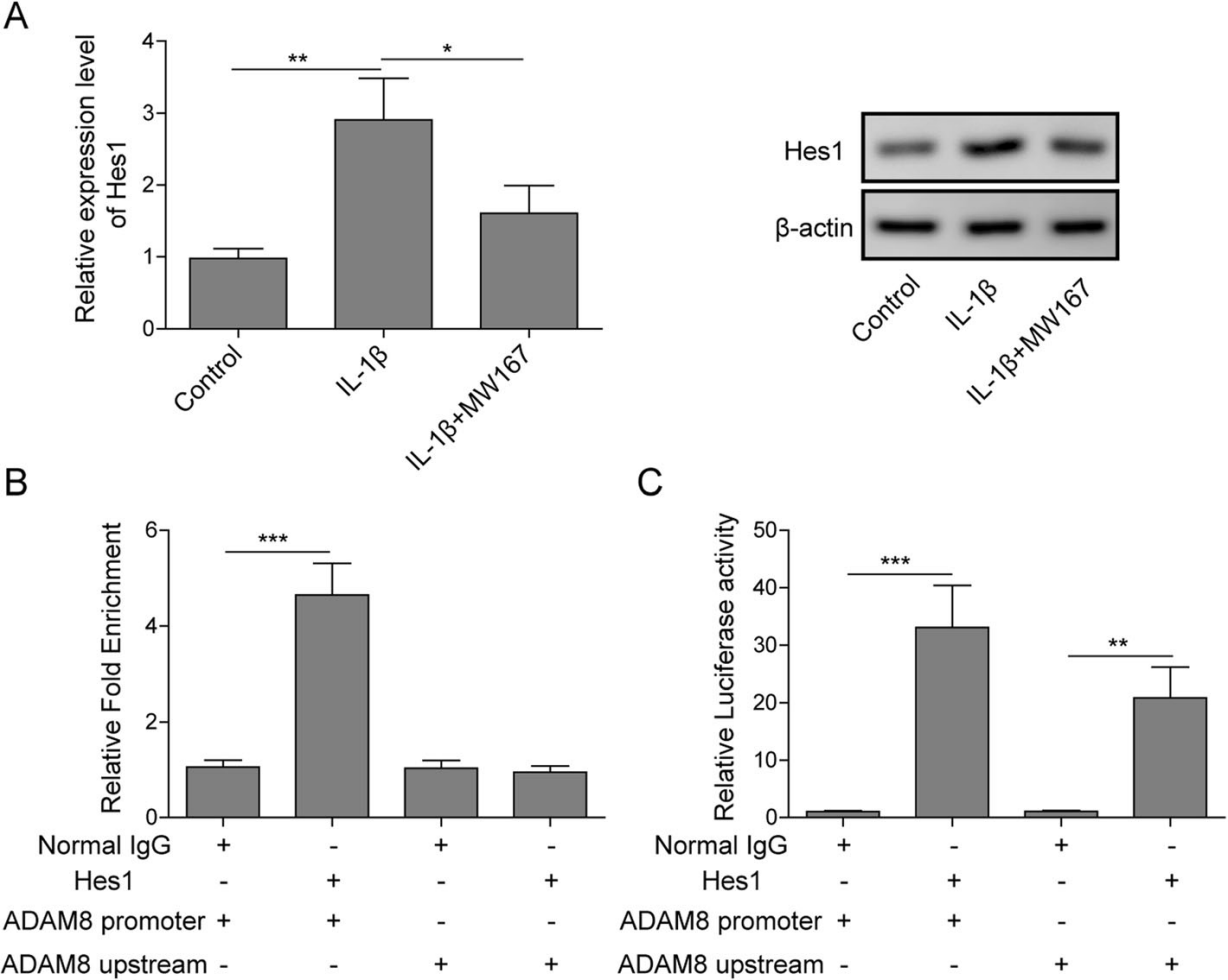
Notch signaling regulated the gene expression of ADAM8 directly via Hes1. **a**: Hes1 mRNA level was stimulated by IL-1β, however, was blocked by MW167. **b**: results of CHIP assays indicated that Hes1 bound to the promoter of ADAM8. **c**: results of luciferase assays indicated that Hes1 stimulated ADAM8 promoter mediated luciferase activity

For a deeper insight to the mechanism of Notch1 mediated stimulation of ADAM8 gene expression, we proposed that Hes1, the downstream effector of Notch1 signaling is involved in this regulation. As shown in Fig. 5a, Q-PCR results revealed that the expression of Hes1 was stimulated by IL-1β, however, was blocked by Notch specific inhibitor MW167, suggesting that Notch1-Hes1 pathway was activated by IL-1β in chondrocytes. Results of Western blotting indicated that Hes1 protein was stimulated by IL-1β, however can be blocked by MW167. To investigate whether Hes1 regulates the expression of ADAM8, a chromatin immunoprecipitation (ChIP) assay and a luciferase assay were performed. As shown in Fig. 5b, the DNA co-immunoprecipitated by anti-Hes1 antibody was detected as positive of ADAM8 promoter via PCR amplification, suggesting that Hes1 was binding to ADAM8 promoter. As shown in Fig. 5c, compared to the control, the chondrocytes transfected with pGL3-ADAM8 promoter plasmid exhibited stimulated luciferase activity, however, this promotion of luciferase activity was notably attenuated by Hes1 shRNA, suggesting that the knockdown of Hes1 resulted in a suppression of ADAM8 transcriptional activity.

### Notch1-ADAM8 positive feedback loop promoted OA phenotype in vitro

**Fig. 6:**
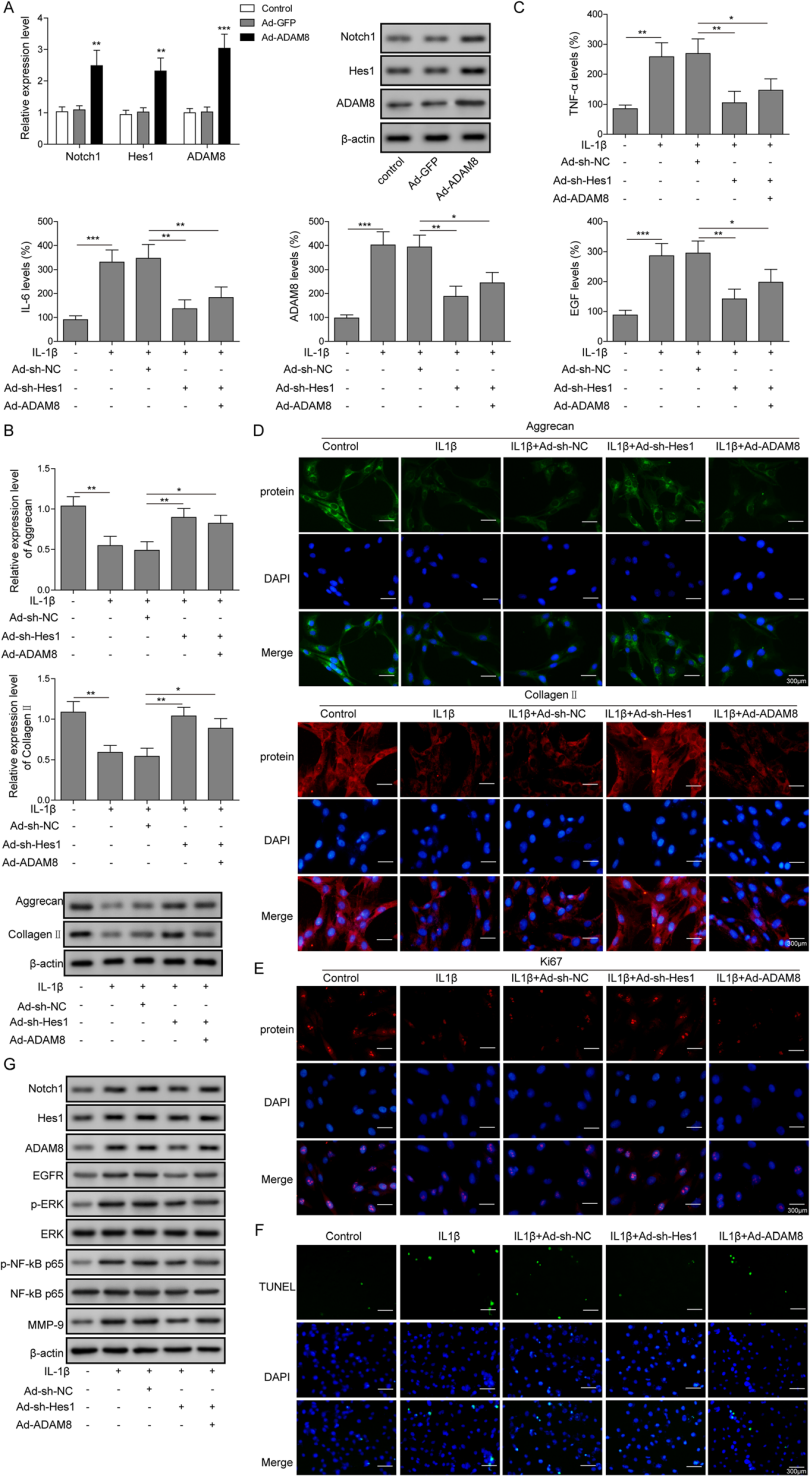
Notch1-ADAM8 positive feedback loop promoted OA phenotype in vitro. **a**: Q-PCR and Western blotting results indicated that overexpression of ADAM8 in chondrocytes stimulated the expression of Notch1 and Hes1. **b**: the level of the secreted form of TNF-α, IL-6, ADAM8 and EGF were stimulated by IL-1β, however, were blocked by the knockdown of Hes1. **c**: Western blotting and **d**: immunofluorescence results indicated that, the protein level of Aggrecan and Collagen II by the knockdown of Hes1, however, were blocked by ADAM8 overexpression. **e**: the protein level of ki67 was stimulated by the knockdown of Hes1, however, was blocked by the overexpression of ADAM8. **f**: cell apoptosis of chondrocytes was suppressed by the knockdown of Hes1, however, was promoted again by the overexpression of ADAM8. **g**: the protein level of Notch1, Hes1, ADAM8, EGFR, p-ERK/ERK, p-NF-kB p65/NF-kB p65 and MMP-9 were suppressed by the knockdown of Hes1, however, were rescued by ADAM8 overexpression

In this section of study, we surprisingly identified that Notch1 and ADAM8 form a positive feedback loop. As shown in Fig. 6a, the overexpression of ADAM8 resulted in a stimulation of both Notch1 and ADAM8 gene expression, as revealed by Q-PCR. Additionally, the protein level of both Notch1 and ADAM8 were found to be stimulated by ADAM8 overexpression. As shown in Fig. 6b, the protein level of TNF-α, IL-6, ADMA8 and EGF were stimulated by IL-1β, however, this stimulative effect was diminished by the knockdown of Hes1 and was rescued by the overexpression of ADAM8. As shown in Fig. 6c, Q-PCR results revealed that, the mRNA level of Collagen II and Aggrecan were suppressed by IL-1β, however, this suppressive effect was diminished by the knockdown of Hes1 and was rescued by the overexpression of ADAM8. This regulation of Collagen II and Aggrecan proteins by IL-1β, Hes1 and ADAM8 were confirmed by western blotting. As shown in Fig. 6d, the regulatory effects mediated by IL-1β, Hes1 and ADAM8 were detected by immunofluorescence assays, and were found to be consistent with the Q-PCR and western blotting results in Fig. 6c. As shown in Fig. 6e, immunofluorescence assays revealed that the expression of Ki-67 was suppressed by IL- 1β and was found to be rescued by the knockdown of Hes1, and was suppressed again by the overexpression of ADAM8. As shown in Fig. 6f, the cell apoptosis of chondrocytes was induced by IL-1β, however, was blocked by the knockdown of Hes1, and was again rescued by the overexpression of ADAM8. As shown in Fig. 6g, the protein level of Notch1, Hes1, p-ERK1/2, p-p65 and MMP-9 were all found to be stimulated by IL-1β, however, this IL-1β mediated stimulations were all attenuated by the knockdown of Hes1, and again were found to be recovered by the overexpression of ADAM8. A summary of the data from Fig. 6b to g suggested that, the IL-1β induced OA phenotype of chondrocytes was reduced by a gene silence of Hes1, and was re-induced by the overexpression of ADAM8, demonstrated that the Notch1/Hes-ADAM8 positive feedback loop facilitated the development of in vitro osteoarthritic phenotype.

### Notch1-ADAM8 positive feedback loop promoted the progression of OA in rat models

**Fig. 7:**
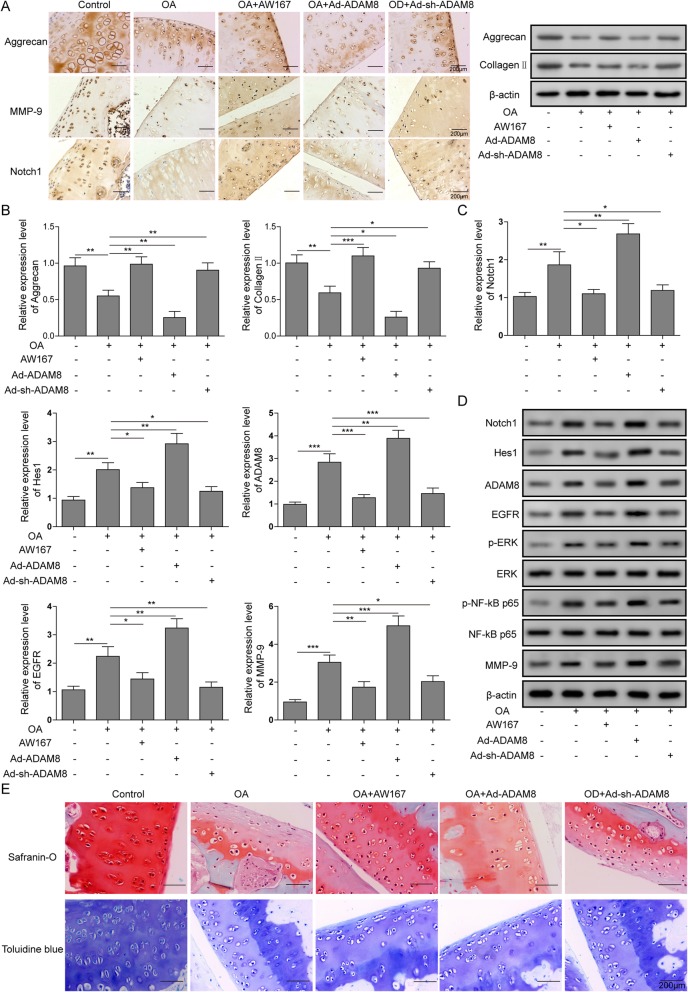
Notch1-ADAM8 positive feedback loop promoted the progression of OA rat models. **a** and **b**: In the OA animal model, the amount of Aggrecan and Collagen II were lower than the control and were rescued by MW167 or knockdown of ADAM8. The lowest content of Aggrecan and Collagen II were found in the ADAM8 overexpression OA animal model. **c**: in OA cartilage, the mRNA level of Notch1, Hes1, ADAM8, EGFR, p-ERK, p-NF-kB p65 and MMP-9 were up-regulated and were blocked by the treatment of MW167 or knockdown of ADAM8. Additionally, the overexpression of ADAM8 presented highest level of Notch1, Hes1, ADAM8, EGFR, p-ERK, p-NF-kB p65 and MMP-9. **d**: in OA cartilage, the protein level of Notch1, Hes1, ADAM8, EGFR, p-ERK, p-NF-kB p65 and MMP-9 were up-regulated and were blocked by the treatment of MW167 or knockdown of ADAM8. Additionally, the overexpression of ADAM8 presented highest level of Notch1, Hes1, ADAM8, EGFR, p-ERK, p-NF-kB p65 and MMP-9. **e**: toluidine blue staining results indicated that the content of glycoprotein in OA cartilage was significantly lower than the control, however, was rescued by MW167 or knockdown of ADAM8. Additionally, the overexpression of ADAM8 presented lowest content of glycoprotein

To confirm the regulatory role of Notch1-ADAM8 positive feedback loop in the development of OA in vivo, a rat OA joint animal model was established. Based on this animal model, rats were treated with MW167 for the inhibition of Notch signaling, with Adv-shADAM8 for the knockdown of ADAM8, or with Adv-ADAM8 for the overexpression of ADAM8, respectively. As shown in Fig. 7a, immunohistochemistry (IHC) results revealed that the protein level of Collagen II and Aggrecan were significantly down-regulated in OA rats, compared to the control. However, the level of the two proteins were rescued by the treatment of Notch inhibitor MW167 or Adv-shADAM8. Furthermore, it was found that the OA rats treated with Adv-ADAM8 resulted in a further suppression of Collagen II and Aggrecan, significant lower level than in OA group. These results were consistent with the mRNA level of Collagen II and Aggrecan in the treatment of MW167, Adv-shADAM8 or Adv-ADAM8 respectively. As shown in Fig. 7b, the mRNA level of Collagen II and Aggrecan were promoted by MW167 or Adv-shADAM8 and were suppressed by Adv-ADAM8. As shown in Fig. 7c, Q-PCR results indicated that the gene expression of Notch1, Hes1, ADAM8, EGFR and MMP-9 were up-regulated in OA animal model. The expression of these genes was suppressed by MW167 or Adv-shADAM8, however, were promoted significantly by Adv-ADAM8. As shown in Fig. 7d, the protein level of Notch1, Hes1, ADAM8, EGFR, MMP-9, p-ERK1/2 and p-p65 were all up-regulated in OA rats, compared to the control. These proteins were suppressed by MW167 or Adv-shADAM8, however, were promoted significantly by Adv-ADAM8. As shown in Fig. 7e, the glycoprotein in OA cartilage was detected by Safranin-O staining and Toluidine blue staining. Our results revealed that the amount of glycoprotein was reduced in OA rats, compared to the control. Furthermore, the glycoprotein level was promoted by the treatment of MW167 or Adv-shADAM8 and was suppressed by Adv-ADAM8. A summary of these data suggested that, the inhibition of Notch signaling or ADAM8 knockdown led to a reduction of OA phenotype, and ADAM8 overexpression led to a promotion of OA phenotype, in the in vivo OA animal model. Additionally, the inhibition of Notch signaling or ADAM8 knockdown resulted in a suppression of EGFR-ERK/ NF-κB signaling pathway, and ADAM8 overexpression led to a stimulation of EGFR-ERK/ NF-κB signaling pathway.

## Discussion

As a member of the A Disintegrin and Metalloproteinase family (ADAM), ADAM8 has been studied extensively in the area of cancer biology [42]. As a type-I transmembrane (TM) glycoprotein, ADAM8 contains a N-terminal prodomain, a metalloproteinase domain, a DIS/cysteine-rich/EGF-like domain and a transmembrane domain, as well as a cytoplasmic tail [43]. As a metalloproteinase, ADAM8 cleaves proteins, and a broad range of proteins were identified as its substrates. ADAM8 exhibits autocatalytic activity and cleaved itself [44]. ADAM8 catalyzes the cleavage reaction of the substrate collagen I, contributing to the invasion, migration and metastasis of cancer cells [25, 28]. Furthermore, ADAM8 was found to cleave fibronectin, contributing to the development of osteoarthritis [28] . However, more details of the regulation of OA by ADAM8 are still to be elucidated. In the present study, our experimental results indicated that the expression of ADAM8 was notably stimulated, suggesting that ADAM8 may play a critical role in OA progression. This result is consistent with previous reports [28] . Moreover, the knockdown of ADAM8 by gene silence in chondrogenic OA cell model resulted in significantly reduction of OA phenotype, including the suppression of MMP-9, TNF-α, IL-6 expression, the restore of Collagen II, Aggrecan expression, the stimulation of cell proliferation, and the inhibition of cell apoptosis. These results suggested that ADAM8 exerts a comprehensive regulation of OA development, including the production of inflammatory factors, ECM, and MMP-9, as well as proliferation and apoptosis. These results demonstrated that the roles of ADAM8 in OA may be much more than only a metalloproteinase for the degradation of ECM. This is consistent with the studies of ADAM8 in tumorigenesis, as reported previously, ADAM8 plays a variety of roles to modulate the activities of cancer cells [42].

One of the most important activities of ADAM8 is the ectodomain shedding of receptors or ligands which were anchored on cell surface [45]. The cleavage of the receptors from cell surface by ADAM8 resulted in a releasing and promotion of soluble form of receptors, and a suppression of respective intracellular signaling via a blocking of specific ligands. For example, ADAM8 mediated the shedding of TNF-R1 in neuron, resulted in a boost of released TNF-R1. This kind of TNF-R1 exerted a neutralizing effect for the ligand TNF-α, and a blocking of cell signaling that induced by TNF-α [46]. Oppositely, the shedding of membrane anchored ligands resulted in a promotion of released ligand proteins, which transactivate specific cell signaling. The shedding of EGFR ligands from cell membrane by ADAMs has been studied extensively. In 2000, Sho Tokumaru *et. al* indicated that, the shedding of EGFR ligands, like the ligand heparin-binding EGF-like growth factor (HB-EGF), led to the release of EGF and stimulation of keratinocyte migration to facilitate wound healing [47]. In 2003, Le Gall SM *at. al* indicated that the ectodomain shedding of full-length pro-EGF was mediated by an unknown metalloprotease [48]. In 2007, U. Sahin pointed out that the shedding of EGFR ligand epigen was mediated by ADAM17 [49]. In 2010, A. Murthy *et.al* indicated that ADAM17 mediated ectodomain shedding of EGFR ligands TGF-a, amphiregulin, and HB-EGF, and dictated hepatocyte apoptosis by a modulation of JNK, ERK and NF-κB signaling [50]. In the present study, we provided evidence to prove that the expression of ADAM8 was stimulated in OA chondrocytes, and the overexpression of ADAM8 was found to stimulate the protein level of EGF. These results suggested that there is an ectodomain shedding of membrane anchored EGF precursor, and the overexpression of ADAM8 resulted in a promotion of soluble EGF, implying that ADAM8 mediated a release of EGF ligand. Distinct from the previous reported ADAM17, our results demonstrated that ADAM8 should be a sheddase. Moreover, the overexpression of ADAM8 resulted in a promotion of ERK1/2 and NF-κB signaling pathway, additionally, the ADAM8 mediated promotion of MMP-9 expression was found to be ERK1/2 and NF-κB signaling dependent, suggesting that ADAM8 activated ERK1/2 and NF-κB to promote MMP-9 expression. Due to the fact that the binding of EGF ligand to EGFR can activate ERK1/2 and NF-κB in various cell types [40, 51, 52], thus we deduced that ADAM8 activated ERK1/2 and NF-κB via the shedding and release of EGF ligand. The released EGF transactivate EGFR and the downstream ERK1/2 and NF-κB signaling. In summary, in this section of study, we identified a new mechanism of ADAM8 for the regulation of OA. ADAM8 promotes the level of EGF ligand, this growth factor activates ERK1/2 and NF-κB to promote MMP-9 gene expression, which in turn facilitates the degradation of ECM and OA progression.

Notch signaling was previously reported to exert crucial role in the regulation of OA [53, 54]. Notch was found to mediated cartilage degradation, fibrosis, and osteoarthritis progression by activating the interleukin-6 (IL-6)-signal transducer and activator of transcription 3 (STAT3) and mitogen-activated protein kinase signaling pathways [55]. Moreover, Notch activate its downstream signaling protein Hes1 to mediate OA-related protein expression. The cartilage tissue specific gene deletion of Hes1 in chondrocytes resulted in reduced OA phenotypes [33]. Luciferase assays indicated that Hes1 directly bound to the promoter of ADAMTS5 and stimulated its gene expression [33]. Additionally, Hes1 was found to stimulate the expression of MMP-13 [33]. In the present study, our results suggested that the inhibition of Notch signaling led to a suppression of the OA phenotype, including the reduction of ADAM8 and MMP-9 expression, the promotion of Collagen II and Aggrecan expression, the promotion of proliferation of chondrocytes, as well as the suppression of apoptosis of chondrocytes. These results are consistent with previous studies, and meanwhile demonstrated a regulatory effect of ADAM8 expression by Notch signaling. Next, we provided evidence to prove that, Hes1, the downstream component of Notch signaling pathway, can regulate the expression of ADAM8. The results of ChIP assays demonstrated that Hes1 can bind to the promoter region of ADAM8. The results of luciferase assays demonstrated that Hes1 directly bind to the promoter of ADAM8 to facilitate its gene transcription. For the first time, our results indicated that ADAM8 is regulated directly by the Notch signaling component Hes1 in chondrocytes. Therefore, a Notch-Hes1-ADAM8 regulatory axis in OA chondrocytes was discovered. Next, we sought to find out the role of ADAM8 in the regulation of Notch signaling. Our results indicated that the overexpression of ADAM8 stimulated the expression of both Notch1 and Hes1, suggesting that ADAM8 can trans- activate Notch signaling pathway. In summary, a Notch-ADAM8 positive feedback loop was identified in OA chondrocytes.

Next, we sought to confirm the role of Notch-ADAM8 positive feedback loop in the pathophysiology of OA, both in vitro and in vivo. Our results indicated that, the knockdown of Hes1 in OA chondrocytes suppressed the OA phenotypes significantly, characterized by the expression of TNF-α, IL-6, Collagen II and Aggrecan, as well as cell proliferation and apoptosis. Additionally, the ADMA8 expression and ADAM8 associated EGF-ERK/NF-κB signaling was also found to be inhibited. Based on the knockdown of Hes1, an overexpression of ADMA8 was performed in OA chondrocytes. Our results indicated that, ADAM8 overexpression can reverse all the changes of OA phenotypes mediated by Hes1 knockdown. These results suggested that the interruption of Notch signaling resulted in a suppression of OA, however the overexpression of ADAM8 reversed this suppressive effect, demonstrating that Notch-ADAM8 positive feedback loop facilitated the progression of OA. The studies in OA rat animal model revealed that the inhibition of Notch signaling or knockdown of ADAM8 led to suppression of OA phenotypes, including promotion of Collagen II and Aggrecan expression, down-regulation of Notch1, Hes1, ADAM8 and MMP-9 expression, as well as EGF-ERK/NF-κB signaling. Importantly, the glycoprotein in the joint cartilage was found to be promoted by Notch inhibition or ADAM8 knockdown. Oppositely, the overexpression of ADAM8 resulted in promotion of OA phenotypes in OA rats, including suppression of Collagen II, Aggrecan and glycoprotein level, and suppression of Notch1, Hes1, ADAM8 and MMP-9 expression, as well as EGF-ERK/NF-κB signaling. Therefore, these results demonstrated that Notch-ADAM8 positive feedback loop facilitated OA progression in rat animal model.

## Conclusions

Collectively, the present study provides evidence to prove that Notch–Hes1 signaling and ADAM8 formed a positive feedback loop to promote the progression of OA. Additionally, ADAM8 facilitated the progression of OA by stimulating EGF-ERK/ NF-κB signaling, probably via the ectodomain shedding of EGF ligands on cell membrane. These findings provide new mechanism for the pathophysiology of OA and suggest ADAM8 as a new therapeutic target for the treatment of OA.

## Data Availability

All data generated or analyzed during this study are included in this published article [and its supplementary information files].

## Abbreviations

ADAMTS: Adamalysin with Thrombospondin Motifs
ECM: Extracellular matix
ICD: Notch-intracellular domain
MMPs: Matrix metalloproteinases
OA: Osteoarthritis
p-ERK1/2: Phosphorylated-ERK1/2
p-p65: Phosphorylated-p65
